# Perspective for Studying the Relationship of miRNAs with Transposable Elements

**DOI:** 10.3390/cimb45040204

**Published:** 2023-04-05

**Authors:** Rustam Nailevich Mustafin, Elza Khusnutdinova

**Affiliations:** 1 Department of Medical Genetics and Fundamental Medicine, Bashkir State Medical University, 450008 Ufa, Russia; 2 Ufa Federal Research Centre, Institute of Biochemistry and Genetics, Russian Academy of Sciences, 450054 Ufa, Russia

**Keywords:** aging, cancer, database, diagnostics, idiopathic pulmonary fibrosis, miRNA, non-coding RNAs, targeted therapy, transposons, transposon-derived miRNA

## Abstract

Transposable elements are important sources of miRNA, long non-coding RNAs genes, and their targets in the composition of protein-coding genes in plants and animals. Therefore, the detection of expression levels of specific non-coding RNAs in various tissues and cells in normal and pathological conditions may indicate a programmed pattern of transposable elements’ activation. This reflects the species-specific composition and distribution of transposable elements in genomes, which underlie gene regulation in every cell division, including during aging. TEs’ expression is also regulated by epigenetic factors (DNA methylation, histone modifications), SIRT6, cytidine deaminases APOBEC3, APOBEC1, and other catalytic proteins, such as ERCC, TREX1, RB1, HELLS, and MEGP2. In evolution, protein-coding genes and their regulatory elements are derived from transposons. As part of non-coding regions and introns of genes, they are sensors for transcriptional and post-transcriptional control of expression, using miRNAs and long non-coding RNAs, that arose from transposable elements in evolution. Methods (Orbld, ncRNAclassifier) and databases have been created for determining the occurrence of miRNAs from transposable elements in plants (PlanTE-MIR DB, PlaNC-TE), which can be used to design epigenetic gene networks in ontogenesis. Based on the data accumulated in the scientific literature, the presence of 467 transposon-derived miRNA genes in the human genome has been reliably established. It was proposed to create an updated and controlled online bioinformatics database of miRNAs derived from transposable elements in healthy individuals, as well as expression changes of these miRNAs during aging and various diseases, such as cancer and difficult-to-treat diseases. The use of the information obtained can open new horizons in the management of tissue and organ differentiation to aging slow down. In addition, the created database could become the basis for clarifying the mechanisms of pathogenesis of various diseases (imbalance in the activity of transposable elements, reflected in changes in the expression of miRNAs) and designing their targeted therapy using specific miRNAs as targets. This article provides examples of the detection of transposable elements-derived miRNAs involved in the development of specific malignant neoplasms, aging, and idiopathic pulmonary fibrosis.

## 1. Introduction

Multicellular eukaryotes are characterized by the expression of a wide variety of non-coding RNAs (ncRNAs), the number of which is several times higher than the number of protein-coding genes [1]. In evolution, the emergence of ncRNAs was due to the protection of genomes from the expression of transposable elements using the RNA interference system (RNAi). This system includes the enzymes Dicer (ribonuclease III), RNA-dependent RNA polymerase (RdRP), and Argonaute-PIWI [2]. These enzymes process transposable elements’ (TEs) transcripts with the formation of small ncRNAs, which are then used by them as guides for silencing the expression of specific TEs [3]. Silencing is carried out due to the complementarity of the nucleotide sequences. In evolution, TEs became sources of regulatory sequences and a large number of protein-coding genes [4], which also become targets for RNAi since they contain complementary sequences for ncRNA. 

The most known ncRNAs are miRNAs—single-stranded molecules (containing about 22 nucleotides) that regulate gene expression by binding to mRNA [5]. In addition, miRNA control is also possible at the transcriptional level due to RNA-directed DNA methylation (RdDM). This phenomenon has long been considered a specific feature of plants only [6]. However, recent studies have described RdDM in humans [7]. Since the RNAi system influences the formation of heterochromatin [8], and miRNAs are able to guide not only DNA methyltransferases, but also histone deacetylases to specific chromosomal loci [6,9], it can be argued that ncRNAs are drivers of epigenetic regulation. However, the higher-order control elements that regulate the features of ncRNA expression are species-specific transposable elements distributed in the genomes [10], which serve as key sources of ncRNA in plants [11] and animals [12]. In addition to epigenetic control using miRNAs, TEs create the global genomic network for in cis regulation of adjacent genes [3]. TEs make up 45% of the human genome [12,13].

**Figure 1:**
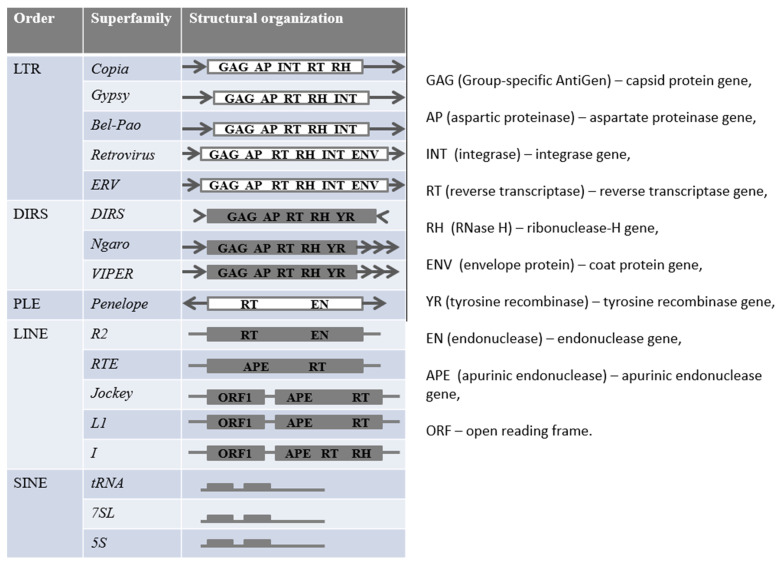
Classification of retroelements.

TEs are classified into retroelements (REs) and DNA-transposons (which change their location by the “cut and paste” or “rolling circle” mechanism). Transposition of REs occurs by reverse transcription of their mRNA and insertion of cDNA into a new locus. REs are subdivided into long terminal repeats containing (LTR-REs) and not containing them (non-LTR-REs) [1]. Non-LTR-REs include the orders DIRS (Dictyostelium discoideum retroelements), PLE (Penelope-like), LINE (long interspersed nuclear elements), and SINE (short interspersed nuclear elements). Each of these orders is classified into several superfamilies (Figure 1) which differ in structural features and mechanisms of movement. For example, PLEs encode an endonuclease (EN—endonuclease), similar to group II introns, and reverse transcriptase (RT—reverse transcriptase), similar to eukaryotic telomerase [13]. Modern online resources are used to classify miRNAs and predict their potential targets, such as mirDeep (for animals), Shortstack, mirDeep-P, mirPlant, miRA, PIPmiR, miR-PREFeR, and miRCat2 (for plants) [14]. 

In addition to miRNAs, noncoding RNAs also include tRNAs (73–93 nucleotides), small nuclear RNAs (150 nucleotides, denoted by the letter U, participate in splicing), small nucleolar RNAs (60–170 nucleotides, necessary for processing ribosomal RNA), Vault-RNA (100 nucleotides, regulate autophagy and apoptosis), Y-RNA (about 100 nucleotides, bind to the Ro60 protein), small NF90-associated RNAs (snaRs—117 nucleotides, involved in translation control), ribosomal RNAs (rRNAs), long non-coding RNAs (more than 200 nucleotides in size), and circular RNA (formed during splicing from exons or introns of mRNA genes) [5]. A common class of ncRNAs are small interfering RNAs (siRNAs) which are generated by the degradation of exogenous dsRNAs, transcribed from TEs or from other types of inverted repeats [2]. Animals are also characterized by the class of piRNAs, 21–35 nucleotides long, which are involved in the regulation of gene expression, antiviral response, and TEs silencing (by targeting histone modifications and DNA methylation) [15]. In humans, ncRNA designations are approved by the HUGO Gene Nomenclature Committee (HGNC). For each gene, www.genenames.org (accessed on 20 December 2022) provides information on its symbol, name, chromosomal localization, and links to key resources, such as Ensembl, NCBI Gene, and GeneCards [5]. The study of the relationship of TEs with miRNAs is promising for the use of ncRNAs as tools for correcting TEs dysregulation during aging, in cancer, and in various idiopathic diseases. To implement this, it is necessary to create an extensive, replenished universal online database that allows you to identify their relationship.

## 2. Differences of the Origin of miRNA from Transposons in Plants from Animals

Unlike in animals, plant miRNAs are completely complementary to their target mRNA sequences. Their binding, in most cases, causes mRNA cleavage [2]. Moreover, mRNAs can contain several regions that are complementary to miRNAs. Both stages of miRNA precursor cleavage are carried out in the nucleus using ribonuclease DCL1, after which the miRNA is transported into the cytoplasm by means of Hasty enzyme, which is homologous to the animal Exportin-5 protein [16]. Plants are characterized by a significant variety and number of specific small ncRNAs, which include tasiRNA (trans-acting short interfering RNA), nat-siRNA (natural antisense short interfering RNA), and hc-siRNA (heterochromatic small interfering RNA) [14]. TEs in plants during evolution become sources of both miRNA genes and protein-coding genes’ exons. Due to these processes, epigenetic regulators (miRNAs) and their targets (gene exons) are formed, and transposable elements (miRNA sources) form dynamic gene networks that control protein-coding genes expression. One of the mechanisms by which miRNAs originate from TEs is the formation of inverted repeats, which are transcribed into RNA hairpin structures processed by Dicer-like enzymes [17]. TE-derived miRNAs (TEDmiR) are involved in vital functions, such as stress responses, a barrier to hybridization in plants, and dynamic transformations of heterochromatin during ontogenesis [3].

For the first time in the world in 2007, a study of rice TEs revealed 21 different small ncRNAs formed from MITE (miniature inverted-repeat transposable elements), which are localized in introns and exons of protein-coding genes, EST regions (expressed sequence tag), and intergenic [18]. A total of 12 TEDmiRs in Arabidopsis and 83 TEDmiRs in rice, which also derived from MITE, were described the next year [19]. An analysis of the miRBase and Repbase Update allowed Lorenzetti et al. to create an online resource (http://bioinfotool.cp.utfpr.edu.br/plantemirdb; accessed on 20 December 2022) for the registration of miRNAs derived from TEs—PlanTE-MIR DB [11]. The main sources of miRNAs in plants are LTR-Res since they constitute the bulk of their genomes. For example, LTR-REs in Asparagus officinalis occupy 91% of the total DNA, in Hordeum vulgare—76%, in Allium cepa—58%, in Zea mays—55% [20]. In 2018, an article was published on the creation of the PlaNC-TE database (http://planc-te.cp.utfpr.edu.br; accessed on 20 December 2022), according to which, in 40 plant genomes, 14350 miRNAs originated from TEs [21]. 

The emergence of miRNAs from transposable elements is an important adaptive mechanism of plants, which is necessary for their survival. In the wheat genome, TEs occupy 85% of all nucleotides. Of these, the most prone to domestication into miRNA precursors are MITE. This mechanism of the miRNA generation plays a role in the development of wheat immune responses. Of the 48 miRNA families, 16 have been shown to be derived from TEs [22]. In different rice species, the miR812 family, derived from MITEs, is involved in immunity against fungal infections. These mechanisms involve many genes (such as *ACO3, CIPK10, LRR*) in the 3′- or 5′-UTR, of which MITEs are located [23]. In the tissues of Arabidopsis sporophytes, small RNAs of 21–22 nucleotides in length were identified, which are transcribed by RNA polymerase-IV from TEs genes. These ncRNAs were involved in the regulation of many plant genes [24]. In 2020, Marakli described 17 new TEDmiRs (in addition to those previously described in PlanTE-MIR DB) that are involved in purine, nitrogen metabolism, oxidative phosphorylation, and other critical plant functions [25]. The appearance of such articles makes it possible to expand the understanding of the role of TEs in the emergence of miRNAs and to create more global database systems for determining the mechanisms of epigenetic regulation of plant and animal ontogenesis.

## 3. The Role of Transposons in the Emergence of miRNA in Animals

**Figure 2:**
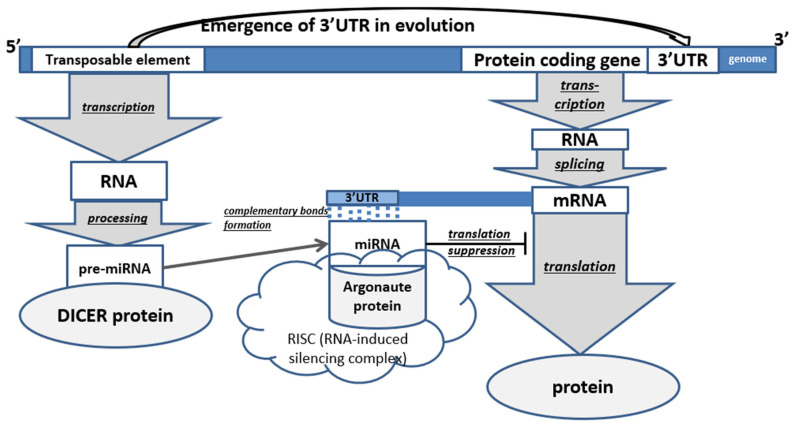
Scheme of the role of transposable elements in post-transcriptional regulation of protein-coding genes in animals (PCG—protein-coding gene). The underlined words in the figure represent processes.

In animals, the main majority of all TEs are non-LTR retroelements (LINE and SINE). In humans, they occupy 35% of all DNA, in mice—28%, in Drosophila—17% [20]. miRNA precursor maturation (by cutting out a parts) is caused by specific enzymes; it initially occurs in the cell nucleus with the help of Drosha ribonuclease-III, after which the RNA is transported into the cytoplasm (with the help of Exportin-5) where it is acted upon by the DICER enzyme [16]. In animals, miRNAs interact with the 3′-UTRs of target mRNAs through partial base pairing (nucleotides 2 to 7 of miRNAs). Binding and interaction of microRNAs with the 3′-UTR lead to the repression of gene expression [2]. 3′-UTR of genes are characterized by the presence of TE residues in them, which form a mutually regulatory system since they become targets for miRNAs derived from their related TEs. As a result, a complex regulatory epigenetic gene network is formed that controls the development of the body (Figure 2) [26].

Evidence for the emergence of miRNAs from TEs in animals has been obtained in numerous studies. For the first time, back in 2005, Smalheiser and Torvik described a model for the formation of miRNAs from TEs sequences in mice, rats, and humans through the formation of hairpin DNA structures between two TEs [27]. In 2006, the results of the analysis of a miRNA cluster on human chromosome 19 were presented, according to which miRNAs are dispersed among Alu-retroelements (referred to as SINE). At least 30 different miRNAs were found to be complementary to Alu [28]. In 2007, 55 different TEDmiRs were described in humans [29]. In 2009, data on 7 TEDmiRs were published in the marsupial *Monodelphis domestiva* [30]. In the same year, 73 miRNAs transcribed from Alu or MIR in humans were characterized using computer modeling. The role of TEs was shown not only as sources of miRNAs, but also as regulators of their expression in time and space during the development of the organism. Retroelements Alu serve as the basis for the transcriptional regulation of certain miRNA genes [10]. Similar data were obtained in the study of piRNA and miRNA derived from TEs at the early stages of embryonic development. These ncRNAs affected the mRNA of genes involved in key pathways in the regulation of embryogenesis (including the *Wnt* and *TGF-β* genes) [31].

In 2010, data on the miR-1302 family, derived from MER53 transposons in humans, were published [32]. In 2011, an article was published about 226 TEDmiR in humans, 141 TEDmiR in mice, and 115 TEDmiR in rhesus monkeys. The authors noted a species-specific expansion of miRNA families, associated with evolutionary transpositions of certain TEs, with large segmental duplications of genomic loci [33]. In 2011, the results of the analysis of more than 15176 individual miRNAs in different animal species were described with the identification of 2392 TEDmiRs [34]. In the same year, a new approach was described for identifying miRNA targets. For this approach, the authors used the analysis of transcripts containing TEs—miRNA precursors. The method was named Orbld (Origin-based identification of miRNA targets). It helped identify targets for 191 TEDmiRs [35]. In 2012, the origin of 182 miRNAs, 788 siRNAs, and 4990 piRNAs from TEs was described in the silkworm [36]. In the same year, the mapping of all miRNA precursors from the miRBASE database, with the determination of the repetitive elements of the genomes overlapping these regions, was reported. The ncRNAclassifier method was developed to classify pre-ncRNAs arising from TEs, and 235 human TEDmiRs and 68 mouse TEDmiRs were described [37]. 

In 2013, genes of 1213 miRNAs in different eukaryotic genomes were studied, of which 1007 (83%) were derived from various TEs (467 from DNA transposons, 235 from LTR-RE, 186 from LINE, 119 from SINE). They identified primate-specific expansions in the miR-151, -378, -6130, -6127, -1260, -548, -4536, and -1273, including 45 human loci [38]. In 2014, using the RepeatMasker program, the GENCODE v.19 database was analyzed and 1900 TEDmiRs were discovered, of which 406 were previously described by other authors [39]. In bats, unlike other animals, DNA-transposons have the highest activity, in which are rich sources of most TEDmiRs. Among all miRNAs, TEDmiRs in bats account for 61%, which is a significant proportion compared to dogs (24%) and horses (17%) [40]. In 2016, data on the detection of 409 TEDmiRs in humans were published [41]. In 2016, an attempt was made to create an MDTE database (miRNAs derived from TEs) of miRNAs derived directly from TEs. Database address: http://bioinf.njnu.edu.cn/MDTE/MDTE.php (accessed on 20 December 2022). This database describes 1251 miRNAs derived from 30 TEs families in humans and 6 animal species (bull, house mouse, chicken, rhesus monkey, common chimpanzee, gray rat) [12]. However, at present, this database is not available, which indicates the relevance of creating a universal online database of miRNAs derived from TEs. 

## 4. Prospects for the Creation of a Human Transposable Elements-Derived miRNA Database

It can be assumed that the majority of animal miRNAs evolved from TEs since transposons are characterized by high mutability during domestication, which causes difficulties in determining the belonging of TEs sequences [42]. Of greatest interest is the study of TEDmiRs in humans, which are associated with severe diseases, since this will reveal the key pathways of disease pathogenesis and, in the future, design targeted methods using ncRNAs targeting TEs. For example, in 2020, a bioinformatic analysis was carried out to find such TEDmiRs using the TransmiR v.2.0 database. A total of 51 specific miRNAs derived from TEs were identified, of which 34 are associated with various human pathologies [43]. Indeed, miRNAs are promising targets for the targeted therapy of various diseases, which is especially important for malignant neoplasms and idiopathic diseases (when the etiology and pathogenesis have not yet been established). In this regard, on the basis of the data presented in the scientific literature by various authors [12,29,32,33,35,37,39,41,43], data were collected on 467 miRNAs derived from transposable elements (Table 1). miRNAs are used to predict tumor formation and outcome. For this, appropriate bioinformation systems are used, such as OncomiR, an online resource for changes in miRNA regulation in malignant neoplasms, which is freely available at www.oncomir.org (accessed on 20 December 2022) [44]. Analysis of this resource using 467 miRNAs derived from transposable (Table 1) allowed me to identify 52 TEDmiRs, in which changes in the expression are characteristic of specific types of malignant tumors [44]. In order to find aging-associated microRNAs derived from transposons, a search was made for the association of 52 TEDmiRs, associated with cancer, with aging in the databases Scopus, WoS, and NCBI. I introduced phrases of specific miRNAs with the words “aging”, “change with age”, “senescence”, and “consenescence” into the search line.

Pathological activation of TEs is characteristic of both human aging and the development of malignant neoplasms, while aging is a risk factor for most types of cancer [45]. Therefore, the scientific literature was analyzed to search for an association with aging of the 52 TEDmiRs, the expression of which changes in malignant neoplasms. This would allow finding common epigenetic relationships between cancer and aging. In the long term, the results obtained could become the basis for a targeted effect on the mechanisms of aging in order to prevent the development of cancer. In my search, 16 of the 52 TEDmiRs (miR-151a, miR-192, miR-211, miR-28, miR-31, miR-335, miR-340, miR-378a, miR-450b, miR-487b, miR-495, miR-511, miR-576, miR-585, miR-708, miR-885) analyzed were found to be associated with aging (Table 1). Aging is characterized by a significant decrease in the level of miR-151a in the blood of healthy people [46], while the expression of miR-192 in the kidneys is significantly increased [47]. Comparison of centenarians with people from families with low life expectancy revealed a significant increase in miR-211 expression in centenarians, which was proposed to be used as a biomarker of aging [48]. A significant decrease in the level of miR-28 expression has been shown in the elderly [49]. Increased expression of miR-31 was revealed during replicative aging [50]. This miRNA is a target of histone deacetylators in both malignant neoplasms and aging [51]. The role of miR-335 was identified in human aging and in age-related neurological diseases [52]. Quantitative transcriptional reverse PCR analysis reveals the role of miR-340 in aging [53]. Estrogen-sensitive miR-378a is involved in the aging mechanisms of the human thymus, as confirmed in experiments on mice [54]. Disruption of miR-450b regulation in cellular senescence, caused by endogenous genotoxic stress, was found [55]. The involvement of miR-487b in the aging of skeletal muscle has been determined [56]. MiR-495 induces senescence of mesenchymal stem cells [57], and expression of miR-511 changes during aging of the nervous system [58].

More enrichment of miR-576 was found in blood plasma exosomes of the elderly compared with young people [59]. miR-487b can be used as a target for targeted therapy of aging-related muscle atrophy, which directly interacts with the long ncRNA MAR1 (muscle anabolic regulator 1) [56]. Oxidative stress contributes to aging and the development of cardiovascular and neurodegenerative diseases. It was found that miR-585 regulates the *PARP-1* gene (poly- (ADP-ribose) polymerase 1), the product of which is involved in the repair of oxidatively damaged DNA. Overexpression of this miRNA increases DNA damage and suppresses cell survival [60]. As a result of the study of miRNA expression in Parkinson’s disease, it was proposed to use miR-885 as a biomarker of human aging and cellular senescence [61]. Experiments on mice have shown the role of miR-450b in aging [55], as well as a decrease in miR-511 expression during aging [58]. The study of 521 different miRNAs in 6 strains of mice with different average lifespans revealed a significant association of three miRNAs, including miR-708 [62], whose expression changes in specific human cancers [44].

An aging-associated disease, the etiology and exact pathogenesis of which is still unknown, is idiopathic pulmonary fibrosis, with an average global prevalence of 2–29 per 100,000 population [63]. Aging increases the risk of developing idiopathic pulmonary fibrosis from 4 per 100,000, for people aged 18–34, to 227.2 per 100,000, for people 75 and older. The average age of idiopathic pulmonary fibrosis patients is 66 years [64]. Survival with idiopathic pulmonary fibrosis is about 3 years, and available drugs only slow down the decline in lung function with little or no effect on mortality [65]. Therefore, it is important to search for the mechanisms of etiopathogenesis of idiopathic pulmonary fibrosis, the results of which could become the basis for creating criteria for the diagnosis and targeted treatment of the disease. The scientific literature was analyzed to look for a change in 467 TEDmiRs in idiopathic pulmonary fibrosis, with the result that identified 12 of these TEDmiRs that are associated with IPF [66,67,68,69,70,71]. Moreover, 9 of them (miR-31, miR-326, miR-335, miR-340, miR-374a, miR-487b, miR-493, miR-495, miR-708) are associated with cancer, and miR-31, miR-335, miR-340, miR-387b, miR-495, and miR-708 are associated with both cancer [44] and aging [50,51,52,53,56,57,62] (Table 1).

The limitations in the methodology are due to the need to search for articles in the Scopus, WoS, and NCBI databases, with their careful analysis and interpretation of the results, which are placed in a table in the form of conclusions. Limitations are due to the limited number of articles reporting associations of TEDmiRs with specific diseases and aging. Thus, when searching for aging-associated TEDmiRs, out of 467, only 52 were associated with malignant neoplasms and 12 with idiopathic pulmonary fibrosis. Prospects for creating a database of miRNAs derived from transposable and their role in the development of diseases are due to the possibility of designing targeted therapy. These miRNAs can be used as targets, and anti-miRNA, miRNA mimic, or peptides formed from their pri-miRNAs (called miPEP) can be tools for influencing them. Currently, scientific articles have already described works on the use of such molecules for the development of antitumor therapy. For example, the systemic delivery of anti-miRNA oligonucleotides (AMO) against miR-181a (oncomiR) in nude mice bearing chondrosarcoma xenografts prolonged survival from 23% to 45%, decreased tumor volume by 32% at day 38, restored *RGS16* expression, and decreased MMP activity [72]. Anti-miRNA was applied against miR-21 for triple-negative breast cancer cells, which demonstrated high efficacy for tumor growth inhibition [73]. For pancreatic cancer cells, anti-miRNA-27a inhibited apoptosis and cell growth via Wnt/β-catenin pathway [74]. AMO against miRNA-221, with AMO-loaded exosomes, was described as an effective antitumor tool for colorectal carcinoma [75]. 

miPEP is translated from pri-miRNA, despite having a short reading frame. They are involved in carcinogenesis, like mature miRNAs formed by processing their pri-miRNAs. Since peptides are more stable molecules than miRNAs or AMOs, the use of miPEP is promising in anticancer therapy. For example, miR-200a encodes miPEP-200a and miR-200b encodes miPEP-200b, which suppresses epithelial-to-mesenchymal transition and thus, inhibits prostate cancer cells. MiR-200a and miR-200b are also implicated in epithelial-to-mesenchymal transition [76]. pri-miRNA-155 encodes miPEP155 that suppresses autoimmune inflammation [77]. It should be noted that some miPEP was shown to promote a positive autoregulatory loop. For example, miPEP133 (tumor-suppressor microprotein) is involved in the regulation of the expression of miR-34a [78].

Regulation of the expression of TEs is possible by means of miRNAs (derived from TEs since they are complementary to their sequences) at the transcriptional level. This is possible due to the phenomenon of RdDM [7]. This will increase the lifespan of people since the pathological expression of TEs is the cause of aging [79]. In addition, the use of miRNAs, complementary to specific TEs, will allow regulating their activity in cancer treatment since the role of pathological activation of TEs in carcinogenesis has been proven [80,81]. It is important to note that Tes are sources of lncRNAs that can serve as pri-miRNAs, with the ability to be translated on ribosomes to form peptides and be processed into miRNAs. Moreover, both formed functional molecules are characterized by participation in the same biological reactions. This indicates the importance of studying the relationship of TEs with lncRNAs and miRNAs. For example, lncRNA MIR22HG (activated in response to chemical stress) is transcribed into pri-miRNA-22, which is translated into a 9 kDa peptide involved in the antiviral response [82]. lncRNA MIR497HG is transcribed into pri-miR-497, which is further processed into two mature miRNAs: miR-497 and miR-195. At the same time, pri-miR-497 is translated into miPEP497 with an oncosuppressive function [83].

## 5. Conclusions

The identification of miRNAs derived from TEs is the basis for determining the regulatory mechanisms through which transposons exert global control over the functioning of genomes. It will allow designing possible ways of influencing physiological and pathological processes in the body, which is promising for the development of modern genetics and medicine. Therefore, it is necessary to create a universal, replenished online database of transposon-derived miRNAs. The scientific literature was analyzed and 467 specific transposon-derived miRNAs, which could form the basis for creating such an online database, were found. The analysis of the data presented in Table 1 made it possible to determine 52 different miRNAs derived from transposons, which are associated with specific malignant neoplasms. Moreover, it was found that 16 of these 52 miRNAs (miR-151a, miR-192, miR-211, miR-28, miR-31, miR-335, miR-340, miR-378a, miR-450b, miR-487b, miR-495, miR-511, miR-576, miR-585, miR-708, miR-885) are also associated with aging, 9 are associated with idiopathic pulmonary fibrosis, and 6 of them (miR-708, miR-495, miR-487b, miR-340, miR-335, miR-31) are associated with both malignant neoplasms and aging. Since TEs are involved in the global regulation of various body functions, my results can be further used to develop diagnostic algorithms for the diagnosis and targeted therapy of aging-associated diseases, such as malignant neoplasms and idiopathic pulmonary fibrosis. miRNAs derived from transposable elements or oligonucleotides antisense, as well as specific peptides formed during translation of pri-miRNAs, can be used as tools for such targeted therapy. 

An analysis of the results presented in the table on the origin of miRNAs from transposons in humans showed that miRNAs are most often formed from LINE elements (108 miRNAs) and SINE elements (94 miRNAs) and less often from DNA transposons (64 miRNAs) and LTR-containing retroelements (53 miRNAs). Since, according to the results of the study (Table 1), the main sources of microRNAs in humans are LINE elements, we analyzed the scientific literature on the role of LINEs in the regulation of embryonic development, in which microRNAs play an important role [84,85,86]. In 2000, Wei et al. described the accumulation of multiple LINE1 insertions in human cell cultures [87]. In 2007, Garcia-Perez et al. revealed the accumulation of LINE1 insertions in human embryonic stem cells, which was accompanied by the suppression of the activity of specific genes required for cell differentiation. On the basis of the obtained data, the researchers suggested that TEs control the work of the genome during the growth and development of organisms [88]. Upon activation of LINE1, their proteins are used to mobilize SINEs. In 2011, Macia et al. reported the expression of several subfamilies of Alu elements in undifferentiated human embryonic stem cells. At the same time, activation of LINE1, located within protein-coding genes which indicates their role in the regulation of these genes, was mainly detected [89]. In addition to tissue cultures, consistent transpositions and activation of LINE1, Alu, and SVA have been identified in vivo during early embryogenesis, during tissue differentiation. These changes caused large-scale structural variations in the genomes of experimental animals. In 2004, Prak et al. showed, in transgenic mouse models, that LINE1 can move in vivo during early development [90]. In 2012, organ-specific and stage-specific changes in cell phenotypes were identified in the C57BL/6J mouse line due to structural transformations of their genomes, which were accompanied by changes in the transcriptional activity of certain ERs [91]. Experiments on two-celled mouse embryos have shown that LINE1 is required for the activation of global gene expression during early embryonic development [92]. LINE1 transcripts themselves function as lncRNAs, interacting with KAP1 and Nucleolin, stimulating rDNA gene expression and silencing other genes in a two-cell embryo by suppressing Dux (a transcription factor that controls the two-cell genetic program) [93].

## Figures and Tables

**Table 1:** Human miRNAs derived from transposons and their involvement in aging, carcinogenesis, and idiopathic pulmonary fibrosis.

№	miRNA Designation	Transposon, miRNA Source [Author]	Diseases and Changes (Increased—↑; Decreased—↓) in miRNA Expression (Expressed Tissue) [Author]	miRNA Expression in Aging (Increased—↑; Decreased—↓) (Expressed Tissue) [Author]
(1)	miR-1183	LINE/L2 [37,41]		
(2)	miR-1200	SINE/MIR [33,37,41]		
(3)	miR-1202	LTR/ERV1 [37,41]; LTR/MER52A [43]		
(4)	miR-1205	SINE/MIR [33,37]		
(5)	miR-1246	LTR/ERVL-MaLR [37,41]		
(6)	miR-1249	LINE/L2 [35,37,41]	↑: BLCA, HNSC, KIRC, LUSC, PRAD, STAD, UCEC; ↓: BRCA, COAD, READ, THCA (tumor tissue) [44]	
(7)	miR-1254-1	SINE/Alu [37,41]		
(8)	miR-1254-2			
(9)	miR-1255a	DNA-TE/TcMar-Tigger [37,41]		
(10)	miR-1255b-1			
(11)	miR-1255b-2			
(12)	miR-1256			
(13)	miR-1257	LTR/ERVL-MaLR [37,41]		
(14)	miR-1260b	DNA-TE/TcMar-Tigger [37]		
(15)	miR-1261	DNA-TE/TcMar-Tigger [37,41,43]		
(16)	miR-1263	LTR/ERV1 [37,41]		
(17)	miR-1264	LINE/L2 [33,37,41]		
(18)	miR-1266	SINE/MIR [37,41]	↑: BLCA, BRCA, CHOL, ESCA, KICH, KIRC, KIRP, LIHC, PRAD, STAD, UCEC; ↓: COAD (tumor tissue) [44]	
(19)	miR-1267	LTR/ERVL-MaLR [37,41]		
(20)	miR-1268a	SINE/Alu [37,41,43]		
(21)	miR-1268b			
(22)	miR-1269a	LTR/ERVL [35,37,41]	↑: BLCA, BRCA, HNSC, LIHC, LUAD, LUSC, PRAD, STAD, THCA, UCEC; ↓: CHOL, KICH (tumor tissue) [44]	
(23)	miR-1269b	LTR/ERVL [37,41]		
(24)	miR-1271	LINE/L2 [35,37,41]	↑: BLCA, ESCA, KIRC, LUSC; ↓: BRCA, COAD, KICH, LIHC, LUSC (tumor tissue) [44]	
(25)	miR-1273a	SINE/Alu [37,41,43]		
(26)	miR-1273c			
(27)	miR-1273d			
(28)	miR-1273f			
(29)	miR-1273g			
(30)	miR-1273h			
(31)	miR-1285-1			
(32)	miR-1285-2			
(33)	miR-1289-1	DNA-TE/hAT-Charlie [37,41]		
(34)	miR-1289-2			
(35)	miR-1290	DNA-TE/TcMar-Tigger [37,41]		
(36)	miR-1293	SINE/Alu [33,37,41]		
(37)	miR-1296	LINE/L2 [12]	↑: BLCA, ESCA, LUSC, PRAD, UCEC; ↓: BRCA, COAD, KIRC, LIHC, READ, THCA (tumor tissue) [44]	
(38)	miR-1298	DNA-TE/X24 [41]		
(39)	miR-130a	LINE/L2 [43]		
(40)	miR-130b	SINE/MIR [29]		
(41)	miR-1302-1	DNA/hAT [32,37,39,41,43]		
(42)	miR-1302-10	DNA/hAT [32,37,39,41]		
(43)	miR-1302-11			
(44)	miR-1302-2			
(45)	miR-1302-3			
(46)	miR-1302-4			
(47)	miR-1302-5			
(48)	miR-1302-6			
(49)	miR-1302-7			
(50)	miR-1302-8			
(51)	miR-1302-9			
(52)	miR-1303	SINE/Alu [37,41,43]		
(53)	miR-1304			
(54)	miR-1321	SINE/MIR [37,41]		
(55)	miR-1343	LINE/L2 [12]	↓: IPF (lung tissue) [67]	
(56)	miR-151a	LINE/L2 [29,35,37,43]	↑: BLCA, BRCA, CESC, COAD, ESCA, HNSC, KICH, KIRC, KIRP, LIHC, LUAD, LUSC, PRAD, READ, STAD, UCEC (tumor tissue) [44]	↓ (serum) [46]
(57)	miR-151b	LINE/L2 [37,41,43]		
(58)	miR-1587	LTR/ERVL-MaLR [37,41,43]		
(59)	miR-1825	LINE/L2 [37,41]		
(60)	miR-1911	LTR/Gypsy [33,37,41]	↑: ESCA, HNSC, LUSC, STAD (tumor tissue) [44]	
(61)	miR-192	LINE/L2 [12]	↑: BLCA, BRCA, COAD, KIRC, LUAD, LUSC, PRAD, READ, STAD, UCEC; ↓: CHOL, KICH, KIRP, LICH, THCA (tumor tissue) [44]	↑ (kidney tissue) [47]
(62)	miR-1972-1	SINE/Alu [37,41,43]		
(63)	miR-1972-2			
(64)	miR-2054	DNA-TE/Helitron [37]		
(65)	miR-211	LINE/L2 [12]	↑: KIRC, KIRP, LIHC; ↓: BRCA, HNSC, LUAD (tumor tissue) [44]	↑ (serum) [48]
(66)	miR-2114	LINE/CR1 [37]	↑: BRCA, KIRC, LIHC (tumor tissue) [44]	
(67)	miR-2115	LINE/L1 [37,41]	↑: BRCA (tumor tissue) [44]	
(68)	miR-219-1	LTR/Gypsy [37]		
(69)	miR-224	DNA-TE/MER135 [37,41,43]	↑: CESC, ESCA, HNSC, KIRC, LIHC, LUAD, LUSC, UCEC; ↓: BRCA, KICH (tumor tissue) [44]	
(70)	miR-23c	SINE/tRNA [37]		
(71)	miR-2355	LINE/RTE-BovB [37,41,43]	↑: BLCA, COAD, ESCA, HNSC, KICH, KIRC, KIRP, LIHC, LUAD, LUSC, PRAD, READ, STAD, UCEC; ↓: LIHC, PAAD, THCA (tumor tissue) [44]	
(72)	miR-28	LINE/L2 [29,35,37,43]	↑: HNSC, KIRC, LUAD, LUSC, PRAD; ↓: BRCA, CHOL, COAD, ESCA, PCPG, READ, STAD, THCA (tumor tissue) [44]	↓ (blood) [49]
(73)	miR-2909	LTR/ERVL-MaLR [37,41]		
(74)	miR-302e	SINE/MIR [37,41,43]		
(75)	miR-31	LINE/L2 [12]	↑: BLCA, CESC, HNSC, KIRP, LUAD, LUSC, STAD, THCA, UCEC (tumor tissue) [44]; IPF (lung tissue) [66]; ↓: KICH, KIRC, PRAD (tumor tissue) [44]	↑ endothelial cells [50], (breast tissue) [51]
(76)	miR-302	SINE/MIR [41]	↑: IPF (lung tissue) [66]	
(77)	miR-3116-1	LINE/L2 [37,41]		
(78)	miR-3116-2			
(79)	miR-3118-1	LINE/L1 [37,41,43]		
(80)	miR-3118-2			
(81)	miR-3118-3			
(82)	miR-3118-4			
(83)	miR-3118-5			
(84)	miR-3118-6			
(85)	miR-3133			
(86)	miR-3134			
(87)	miR-3135a	SINE/Alu [37,41]		
(88)	miR-3135b			
(89)	miR-3137	DNA-TE/TcMar-Tigger [37,41]		
(90)	miR-3139	LINE/L2 [37,41]		
(91)	miR-3144	LINE/L1 [37,41,43]	↑: HNSC, KICH (tumor tissue) [44]	
(92)	miR-3149	LINE/L1 [41]		
(93)	miR-3159	SINE/Alu [37,41]		
(94)	miR-3161	DNA-TE/hAT [37]		
(95)	miR-3163	SINE/MIR [37,41]		
(96)	miR-3164	DNA-TE/ TcMar-Tigger [41]		
(97)	miR-3166	LINE/L2 [37,41]		
(98)	miR-3168	SINE/MIR [37,41]		
(99)	miR-3169			
(100)	miR-3174	DNA-TE/hAT-Charlie [37,41]		
(101)	miR-3176	LINE/L1 [37]		
(102)	miR-3179-1	SINE/Alu [33,37,41]		
(103)	miR-3179-2	SINE/Alu [33,37,41]		
(104)	miR-3179-3			
(105)	miR-3185	DNA-TE/hAT [37]		
(106)	miR-3194	SINE/MIR [37,41]	↑: STAD (tumor tissue) [44]	
(107)	miR-3200	ERV-L [35,37,41]	↑: BLCA, BRCA, CHOL, HNSC, KIRP, LIHC, LUSC, STAD, UCEC; ↓: KIRC (tumor tissue) [44]	
(108)	miR-3201	LINE/L2 [37,41]		
(109)	miR-320b-2	LINE/L2 [33,37,41]		
(110)	miR-320c-1	LINE/RTE [37]		
(111)	miR-320c-2	DNA-TE/Ginger2/TDD [37]		
(112)	miR-320d-1	LINE/L1 [43]		
(113)	miR-320d-2	LINE/CR1 [37]		
(114)	miR-325	LINE/L2 [29,37,39,41]		
(115)	miR-326	DNA-TE/hAT-Tip100 [37,41]	↑: BLCA, KIRC, PCPG, UCEC; ↓: BRCA, COAD, KICH, LIHC, LUSC, READ, THCA (tumor tissue) [44]; IPF (lung tissue) [67]	
(116)	miR-330	SINE/MIR [29,37]		
(117)	miR-335	SINE/MIR [33,37,41,43]	↑: BLCA, COAD, ESCA, HNSC, LUAD, LUSC, PRAD, STAD, THCA, UCEC; ↓: BRCA, KICH, KIRC, LIHC (tumor tissue) [44]; IPF (lung tissue) [66]	↑ (brain tissue) [52]
(118)	miR-340	DNA-TE/TcMar [35,37,41,43]	↑: BRCA, COAD, KICH, KIRC, KIRP, LUAD, LUSC, PRAD, UCEC (tumor tissue) [44]; IPF (lung tissue) [70] ↓: CHOL, LIHC, PAAD (tumor tissue) [44]	↑ (serum) [53]
(119)	miR-342	SINE/tRNA-RTE [35,37,41,43]	↑: BLCA, BRCA, CESC, HNSC, KIRC, KIRP, PRAD, STAD, UCEC; ↓: COAD, LIHC, LUAD, PAAD, READ, THCA (tumor tissue) [44]	
(120)	miR-345	SINE/MIR [29,37]		
(121)	miR-361	DNA-TE/hAT [37]		
(122)	miR-3611	DNA/TcMar-Tigger [37,41]		
(123)	miR-3617	SINE/MIR [37,41]		
(124)	miR-3622a	SINE/Alu [12]	↓: LUAD (tumor tissue) [44]	
(125)	miR-3622b	SINE/Alu [37,41]		
(126)	miR-3646	SINE/MIR [33,37]		
(127)	miR-3648	DNA-TE/MER [37]		
(128)	miR-3657	LINE/L1 [37,41]		
(129)	miR-3664	DNA-TE/TcMar-Tigger [33,37,41]		
(130)	miR-3665	DNA-TE/EnSpm [37]		
(131)	miR-3667	LTR/ERVL-MaLR [37,41]		
(132)	miR-3668	SINE/MIR [33,37]		
(133)	miR-3670-1	LTR/ERVL [37,41]		
(134)	miR-3670-2			
(135)	miR-3672	LINE/L1 [37,41]		
(136)	miR-3674	LTR/ERV1 [37,41]		
(137)	miR-3680-1	DNA-TE/hAT-Tip100 [41]		
(138)	miR-3680-2			
(139)	miR-3681	LTR/ERVL [37,41]		
(140)	miR-3686	LINE/L1 [37,41]		
(141)	miR-3689c	LINE/L1 [37]		
(142)	miR-370	SINE/MIR [29,37]		
(143)	miR-3713	DNA/TcMar-Tigger [37,41]		
(144)	miR-374a	LINE/L2 [37,41,43]	↑: BLCA, BRCA, COAD, KIRC, KIRP, PRAD, READ, STAD; ↓: CHOL, HNSC, LUSC (tumor tissue) [44]; ↓: IPF (lung tissue) [66]	
(145)	miR-374b	LINE/L2 [29,37]	↑: BLCA, BRCA, COAD, ESCA, HNSC, KIRC, KIRP, PRAD, STAD, UCEC; ↓: THCA (tumor tissue) [44]	
(146)	miR-374c	LINE/L2 [37,41]		
(147)	miR-378a	SINE/MIR [29,37,43]	↑: PAAD; ↓: BRCA, CHOL, COAD, HNSC, LIHC, LUAD, PAAD, PRAD, READ, STAD (tumor tissue) [44]	↑ (thymus tissue) [54]
(148)	miR-378b	SINE/MIR [37,41,43]		
(149)	miR-378d-1			
(150)	miR-378d-2			
(151)	miR-378e			
(152)	miR-378f			
(153)	miR-378g			
(154)	miR-378h			
(155)	miR-378i	SINE/MIR [37,43]		
(156)	miR-3908	SINE/Alu [37,41]		
(157)	miR-3909	LINE/L2 [12,37]	↑: LIHC (tumor tissue) [44]	
(158)	miR-3910-1	LINE/L1 [37,41]		
(159)	miR-3910-2			
(160)	miR-3912			
(161)	miR-3915			
(162)	miR-3919	LINE/L1 [37]		
(163)	miR-3920	LINE/L2 [33,37,41]		
(164)	miR-3921	LINE/L2 [33,37]		
(165)	miR-3923	LTR/ERVL-MaLR [37,41]		
(166)	miR-3925	DNA-TE/TcMar-Tigger [37,41]		
(167)	miR-3927	LTR/ERVL-MaLR [37,41]		
(168)	miR-3929	SINE/Alu [37,41]		
(169)	miR-3934	SINE/MIR [35,37,41]	↑: BRCA, HNSC, KIRC, LUSC, STAD, UCEC (tumor tissue) [44]	
(170)	miR-3936	LTR/ERVL [33,35,37,41]		
(171)	miR-3937	LTR/ERV3 [37]		
(172)	miR-3972	DNA-TE/hAT-Tip100 [37,41]		
(173)	miR-3973	DNA-TE/TcMar-Tigger [41]		
(174)	miR-3977	DNA-TE/hAT-Tip100 [41]		
(175)	miR-421	LINE/L2 [29,35,37,43]	↑: BLCA, BRCA, ESCA, HNSC, KIRP, LIHC, LUAD, LUSC, STAD, UCEC; ↓: THCA (tumor tissue) [44]	
(176)	miR-422a	SINE/MIR [37,41]		
(177)	miR-4263	LINE/L1 [37,41]		
(178)	miR-4281	LTR/ERV1 [37]		
(179)	miR-4286	LTR/ERV3 [37]		
(180)	miR-4288	LTR/ERVL [33,37,41]		
(181)	miR-4293	SINE/tRNA [37,41]		
(182)	miR-4311	LINE/L2 [41]		
(183)	miR-4317	SINE/MIR [33,37]		
(184)	miR-4418	LINE/L2 [37,41]		
(185)	miR-4419a	SINE/Alu [37,41]		
(186)	miR-4419b			
(187)	miR-4420	SINE/MIR [37,41]		
(188)	miR-4421	LTR/ERV1 [37,41]		
(189)	miR-4422	LTR/Gypsy [37,41]		
(190)	miR-4424	LINE/L1 [37,41]		
(191)	miR-4425	SINE/MIR [37,41]		
(192)	miR-4428	LTR/ERV1 [37,41]		
(193)	miR-4430	SINE/Alu [37,41]		
(194)	miR-4431	LTR/ERVL-MaLR [37,41]		
(195)	miR-4433	LINE/L2 [37,41]		
(196)	miR-4433b			
(197)	miR-4438	LINE/L1 [37,41]		
(198)	miR-4445			
(199)	miR-4447	DNA-TE/hAT-Charlie [37,41]		
(200)	miR-4448	LTR/ERVK [37,41]		
(201)	miR-4452	SINE/Alu [37,41,43]		
(202)	miR-4454	LTR/ERV1 [37,41]		
(203)	miR-4455	LINE/L1 [37,41]		
(204)	miR-4457			
(205)	miR-4459	SINE/Alu [37,41]		
(206)	miR-4460	LTR/ERVL [37,41]		
(207)	miR-4463	DNA-TE/hAT-Charlie [37,41]		
(208)	miR-4468	LINE/R1 [37]		
(209)	miR-4472-2	SINE/Alu [37,41]		
(210)	miR-4477a	DNA-TE/TcMar-Tigger [37,41]		
(211)	miR-4477b			
(212)	miR-4480	SINE/MIR [37,41]		
(213)	miR-4481	LTR/ERVL-MaLR [37,41]		
(214)	miR-4483	SINE/Alu [37,41]		
(215)	miR-4484	LTR/ERV1 [37,41]		
(216)	miR-4491	DNA-TE/hAT-Blackjack [37,41]		
(217)	miR-4494	DNA-TE/TcMar-Tigger [37,41]		
(218)	miR-4495	SINE/MIR [37,41]		
(219)	miR-4496			
(220)	miR-450b	LINE/L1 [33,35,37,41,43]	↑: BLCA, BRCA, COAD, ESCA, HNSC, KIRC, LUAD, LUSC, READ, STAD, THCA; ↓: CHOL, KICH, KIRP, LIHC, PRAD, UCEC (tumor tissue) [44]	↓ (liver tissue) [55]
(221)	miR-4501	SINE/MIR [37,41]		
(222)	miR-4502	DNA-TE/TcMar-Tigger [37,41]		
(223)	miR-4504	LINE/L1 [37,41]		
(224)	miR-4506	SINE/MIR [37,41]		
(225)	miR-4507	DNA-TE/P-1 [37]		
(226)	miR-4508	SINE [37]		
(227)	miR-4510	LINE/L2 [37,41]		
(228)	miR-4512	SINE/Alu [37,41]		
(229)	miR-4518	DNA/hAT-Charlie [37,41]		
(230)	miR-4520a	SINE/MIR [37,41]		
(231)	miR-4520b	SINE/tRNA [37]		
(232)	miR-4525	LTR/ERV1 [37,41]		
(233)	miR-4527	LTR/ERVL-MaLR [37,41]		
(234)	miR-4537	DNA-TE/P-1 [37]		
(235)	miR-4538	DNA-TE/P-1 [37]		
(236)	miR-4640	LTR/Copia [37]		
(237)	miR-4656	SINE/tRNA [37]		
(238)	miR-466	LINE/L1 [37,41,43]		
(239)	miR-4661	LTR/Gypsy [41]		
(240)	miR-4662a	LINE/L1 [37,41]		
(241)	miR-4662b			
(242)	miR-4666b	LTR/ERVL-MaLR [41]		
(243)	miR-4671	SINE/tRNA-RTE [41]		
(244)	miR-4672	SINE/MIR [37,41]		
(245)	miR-4676	LINE/L2 [37,41]		
(246)	miR-4684	DNA-TE/hAT-Charlie [37,41]		
(247)	miR-4699	LINE/L2 [37,41]		
(248)	miR-4703	DNA-TE/TcMar-Tigger [41]		
(249)	miR-4704	LTR/ERVL-MaLR [37,41]		
(250)	miR-4712	SINE-MIR [37,41]		
(251)	miR-4731	LINE/CR1 [37,41]		
(252)	miR-4739	LTR/Gypsy [37]		
(253)	miR-4750	LINE/CR1 [37]		
(254)	miR-4753	LINE/L1 [37,41]		
(255)	miR-4756	DNA/hAT-Tip100 [37,41]		
(256)	miR-4771-1	LINE/L1 [37]		
(257)	miR-4771-2			
(258)	miR-4772	LINE/L1 [37,41]		
(259)	miR-4775	DNA-TE/TcMar-Tc1 [41]		
(260)	miR-4786	LINE/L1 [37,41]		
(261)	miR-4797	SINE/5S-Deu-L2 [41]		
(262)	miR-4800	DNA-TE/Sola [37]		
(263)	miR-4801	SINE/MIR [37,41]		
(264)	miR-487b	SINE/MIR [33,37,41]	↑: LUAD, LUSC (tumor tissue) [44]; IPF (lung tissue) [71]; ↓: BRCA, HNSC, KICH, KIRC, KIRP, LIHC, PRAD, THCA, UCEC (tumor tissue) [44]	↑ (skeletal muscles) [56]
(265)	miR-493	LINE/L2 [29,35,37]	↑: BRCA, ESCA, LUAD, LUSC, READ, STAD (tumor tissue) [44]; IPF (lung tissue) [71] ↓: KICH, KIRC, KIRP, LIHC, PRAD (tumor tissue) [44]	
(266)	miR-495	ERV-L/MaLR [12]	↑: COAD, LUAD, READ (tumor tissue) [44]; IPF (lung tissue) [71]; ↓: BRCA, HNSC, KICH, KIRC, KIRP, LIHC, THCA, UCEC (tumor tissue) [44]	↑ (mesenchymal stem cells) [57]
(267)	miR-4999	LINE/L1 [41]		
(268)	miR-5003	SINE/MIR [41]		
(269)	miR-5007	LTR/ERVL-MaLR [41]		
(270)	miR-5009	DNA-TE/TcMar-Tigger [41]		
(271)	miR-5011			
(272)	miR-502	LINE/L2 [12]	↑: BLCA, LIHC, PRAD, STAD, UCEC; ↓: COAD, KIRC, KIRP, LUSC, PAAD, THCA (tumor tissue) [44]	
(273)	miR-5094	SINE/Alu [41]		
(274)	miR-5095	SINE/Alu [37,41]		
(275)	miR-5096			
(276)	miR-5100	SINE/MIR		
(277)	miR-511	LINE/L1 [12]	↑: HNSC, PRAD, READ, STAD; ↓: BRCA, CHOL, KICH, KIRP, LIHC, LUSC, PCPG (tumor tissue) [44]	↑ (brain tissue) [58]
(278)	miR-513a-1	DNA-TE/MER [37,43]		
(279)	miR-513a-2			
(280)	miR-513b			
(281)	miR-513c			
(282)	miR-517a	SINE/Alu [10]	↓: LUAD (tumor tissue) [44]	
(283)	miR-518d	LINE/RTE [43]		
(284)	miR-520d	SINE/Alu [12]	↑: LIHC (tumor tissue) [44]	
(285)	miR-544a	DNA-TE/hAT-Charlie [37,41,43]		
(286)	miR-544b			
(287)	miR-545	LINE/L2 [29,35,37,43]	↑: BRCA, KIRC, LIHC, READ (tumor tissue) [44]	
(288)	miR-548a-1	DNA-TE/TcMar-Mariner [37,41,43]		
(289)	miR-548a-2			
(290)	miR-548a-3			
(291)	miR-548aa-1			
(292)	miR-548aa-2			
(293)	miR-548ab			
(294)	miR-548ac			
(295)	miR-548ad			
(296)	miR-548ae-1			
(297)	miR-548ae-2			
(298)	miR-548ag-1			
(299)	miR-548ag-2			
(300)	miR-548ah			
(301)	miR-548ai			
(302)	miR-548aj-1			
(303)	miR-548aj-2			
(304)	miR-548ak			
(305)	miR-548al			
(306)	miR-548am			
(307)	miR-548an			
(308)	miR-548ao			
(309)	miR-548ap			
(310)	miR-548aq			
(311)	miR-548ar			
(312)	miR-548as	DNA-TE/TcMar-Mariner [41]		
(313)	miR-548at			
(314)	miR-548au			
(315)	miR-548av			
(316)	miR-548aw			
(317)	miR-548ax			
(318)	miR-548ay			
(319)	miR-548az			
(320)	miR-548b			
(321)	miR-548ba			
(322)	miR-548c			
(323)	miR-548d-1			
(324)	miR-548d-2			
(325)	miR-548e			
(326)	miR-548f-1			
(327)	miR-548f-2			
(328)	miR-548f-3	DNA-TE/TcMar-Mariner [41]		
(329)	miR-548f-4			
(330)	miR-548f-5			
(331)	miR-548g			
(332)	miR-548h-1			
(333)	miR-548h-2			
(334)	miR-548h-3			
(335)	miR-548h-4			
(336)	miR-548h-5			
(337)	miR-548i-1			
(338)	miR-548i-2			
(339)	miR-548i-3			
(340)	miR-548i-4			
(341)	miR-548j			
(342)	miR-548k			
(343)	miR-548l			
(344)	miR-548m			
(345)	miR-548n	DNA-TE/TcMar-Mariner [39,41]		
(346)	miR-548o			
(347)	miR-548o-2			
(348)	miR-548p			
(349)	miR-548q			
(350)	miR-548s			
(351)	miR-548t			
(352)	miR-548u			
(353)	miR-548v			
(354)	miR-548w			
(355)	miR-548x			
(356)	miR-548x-2			
(357)	miR-548y			
(358)	miR-548z			
(359)	miR-549a	SINE/MIR [33,41]		
(360)	miR-551a	LINE/L1 [12]	↑: BRCA, LUAD, LUSC, STAD (tumor tissue) [44]	
(361)	miR-552	LINE/L1 [29,37,41]	↑: LIHC, READ, STAD (tumor tissue) [44]	
(362)	miR-553	SINE/MIR [33,41]		
(363)	miR-558	LTR/ERVL-MaLR [37,39,41]		
(364)	miR-5584	SINE/MIR [41]		
(365)	miR-5585	SINE/Alu [41]		
(366)	miR-5586	LINE/L1 [41]		
(367)	miR-5589	DNA-TE/hAT-Tip100 [41]		
(368)	miR-5590	LINE/L1 [41]		
(369)	miR-5591			
(370)	miR-562	LINE/L1 [37]		
(371)	miR-566	SINE/Alu [37,41]		
(372)	miR-568	DNA-TE/Tc1-Mariner [37]		
(373)	miR-5681a	SINE/MIR [41]		
(374)	miR-5682	LINE/L1 [41]		
(375)	miR-5683	DNA-TE/hAT-Charlie [41]		
(376)	miR-5684	SINE/Alu [41]		
(377)	miR-5689			
(378)	miR-5691	DNA-TE/hAT-Cahrlie [41]		
(379)	miR-5694	LTR/ERVL [41]		
(380)	miR-5695	LTR-ERV1 [41]		
(381)	miR-5697	LINE/L1 [41]		
(382)	miR-5698			
(383)	miR-570	DNA-TE/TcMar-Mariner [37,41,43]		
(384)	miR-5700	LINE/L2 [41]		
(385)	miR-5706	DNA/TcMar-Tigger [41]		
(386)	miR-5708	SINE/Alu [41]		
(387)	miR-571	LINE/L1 [37,41]		
(388)	miR-575	SINE [37]		
(389)	miR-576	LINE/L1 [29,37]	↑: BLCA, BRCA, ESCA, HNSC, KICH, KIRC, KIRP, LUAD, LUSC, PRAD, READ, STAD, UCEC; ↓: CHOL, LIHC, THCA (tumor tissue) [44]	↑ (blood plasma) [59]
(390)	miR-577	LINE/L2 [33,37,41]	↑: BLCA, CHOL, COAD, HNSC, KICH, LUAD, LUSC, READ, STAD, UCEC; ↓: KIRC, KIRP, THCA (tumor tissue) [44]	
(391)	miR-578	LINE/CR1 [37]		
(392)	miR-579	DNA-TE/TcMar-Mariner [37,41]		
(393)	miR-581	DNA-TE/hAT-Charlie [33,37,41]		
(394)	miR-582	LINE/CR1 [29,35,37]	↑: BRCA, COAD, KICH, PRAD, READ; ↓: CHOL, HNSC, LIHC, THCA (tumor tissue) [44]	
(395)	miR-584	DNA-TE/hAT-Blackjack [29,35,37]	↑: BLCA, ESCA, HNSC, KICH, KIRC, KIRP, PRAD, STAD; ↓: BRCA, LUAD, THCA (tumor tissue) [44]	
(396)	miR-585	ERV-L/MaLR [37,41]	↓: BRCA, KICH, KIRC, THCA (tumor tissue) [44]	↑ (endothelial cells) [60]
(397)	miR-587	DNA-TE/hAT [37]		
(398)	miR-588	LINE/L1 [37,41]		
(399)	miR-591	DNA-TE/hAT-Charlie [33,37,41]		
(400)	miR-598	DNA-TE/CACTA LP [37]		
(401)	miR-603	DNA-TE/TcMar-Mariner [37,41,43]		
(402)	miR-606	LINE/L1 [37,41]		
(403)	miR-607	SINE/MIR [29,37,41]		
(404)	miR-608	LINE/L2 [33,37]		
(405)	miR-6088	SINE/Alu [41]		
(406)	miR-612	SINE/MIR [33,37,41]		
(407)	miR-6127	SINE/MIR [41]		
(408)	miR-6130	LINE/L1 [41]		
(409)	miR-616	LINE/L2 [35,37,41]	↑: KICH, KIRC, KIRP, LUSC, UCEC; ↓: CHOL, LIHC (tumor tissue) [44]	
(410)	miR-619	LINE/L1; SINE/Alu [35,37,39,41,43]		
(411)	miR-625	LINE/L1 [35,37,41]		
(412)	miR-626			
(413)	miR-630	SINE/MIR [12]	↓: IPF (lung tissue) [68]	
(414)	miR-6303	DNA-TE/MADE1 [43]		
(415)	miR-633	SINE/MIR [35,37,41]		
(416)	miR-634	LINE/L1 [37,41]		
(417)	miR-637	LINE/L1 [33,37,41,43]		
(418)	miR-638	DNA-TE/hAT [37]		
(419)	miR-640	SINE/MIR [37,41]		
(420)	miR-644a	LINE/L1 [37,41]		
(421)	miR-645	DNA-TE/hAT-Charlie [37,41]		
(422)	miR-646	LTR/ERVL [37,41]		
(423)	miR-649	DNA-TE/TcMar-Tigger [37,39,41]		
(424)	miR-6500	LTR/ERV1 [41]		
(425)	miR-6503	LTR/ERVL-MaLR [41]		
(426)	miR-6507	LINE/L1 [41]		
(427)	miR-652	DNA/hAT-Tip100 [29,35,37,43].	↑: BLCA, ESCA, HNSC, LIHC, STAD, THCA, UCEC; ↓: COAD, KICH, LUAD, LUSC, THCA (tumor tissue) [44]	
(428)	miR-659	DNA-TE/hAT-Tip100 [37,41]		
(429)	miR-663	LINE/I [37]		
(430)	miR-663b	LTR/Gypsy [37]		
(431)	miR-6745	LINE/L2 [41]		
(432)	miR-6839	LTR/ERV1 [41]		
(433)	miR-6854	DNA-TE/hAT-Charlie [41]		
(434)	miR-6887	LINE/L2 [41]		
(435)	miR-708	LINE/L2 [35,37,41]	↑: BLCA, BRCA, CHOL, COAD, HNSC, KIRC, LUAD, LUSC, PRAD, READ, STAD; ↓: KICH, THCA (tumor tissue) [44]; IPF (lung tissue) [69]	↑ (spleen tissue) [62]
(436)	miR-7151	LINE/L1 [41]		
(437)	miR-7157			
(438)	miR-720	LTR/ERV1 [37]		
(439)	miR-769	LINE/CR1 [12]	↑: BLCA, BRCA, ESCA, HNSC, KIRC, KIRP, LIHC, LUSC, PRAD, STAD, UCEC; ↓: COAD (tumor tissue) [44]	
(440)	miR-7702	LINE/RTE-BovB [41]		
(441)	miR-7849	LTR/ERVL-MaLR [41]		
(442)	miR-7850			
(443)	miR-7851	SINE/Alu [41]		
(444)	miR-7853	DNA-TE/hAT-Cahrlie [41]		
(445)	miR-7973-1	SINE/MIR [41]		
(446)	miR-7973-2			
(447)	miR-7975	LTR/ERV1 [41]		
(448)	miR-7977			
(449)	miR-7978	LINE/L1 [41]		
(450)	miR-8056	LINE/L2 [41]		
(451)	miR-8067	LINE/L1 [41]		
(452)	miR-8074			
(453)	miR-8076	SINE/MIR [41]		
(454)	miR-8079	SINE/MIR [41]		
(455)	miR-8084	LINE/L1 [41]		
(456)	miR-8086	SINE/Alu [41]		
(457)	miR-877	LINE/L1 [37]		
(458)	miR-885	SINE/MIR [37,41]	↑: KICH; ↓: CHOL (tumor tissue) [44]	↑ (blood) [61]
(459)	miR-887	LINE/L2 [37,41]	↑: BRCA; ↓: HNSC, KICH, KIRP, PAAD, THCA (tumor tissue) [44]	
(460)	miR-891a	SINE/MIR [33,37,41]		
(461)	miR-891b			
(462)	miR-921	SINE/MIR [37,41]		
(463)	miR-924	LTR/ERV1 [37]		
(464)	miR-941-1	LTR/Gypsy [37]		
(465)	miR-941-3			
(466)	miR-941-4			
(467)	miR-95	LINE/L2 [29,37,43]	↑: CHOL, COAD, PRAD, READ, STAD, UCEC; ↓: HNSC, KICH, PCPG, THCA (tumor tissue) [44]	

List of abbreviations for the table: BLCA—bladder urothelial carcinoma; BRCA—breast invasive carcinoma; CESC—cervical squamous cell carcinoma and endocervical adenocarcinoma; CHOL—cholangiocarcinoma; COAD—colon adenocarcinoma; ESCA—esophageal carcinoma; HNSC—head and neck squamous cell carcinoma; IPF—idiopathic pulmonary fibrosis; KICH—kidney chromophobe; KIRC—kidney renal clear cell carcinoma; KIRP—kidney renal papillary cell carcinoma; LIHC—liver hepatocellular carcinoma; LUAD—lung adenocarcinoma; LUSC—lung squamous cell carcinoma; PAAD—pancreatic adenocarcinoma; PRAD—prostate adenocarcinoma; PCPG—pheochromocytoma and paraganglioma; READ—rectal adenocarcinoma; STAD—stomach adenocarcinoma; THCA—thyroid carcinoma; UCEC—uterine corpus endometrial carcinoma.

## Data Availability

Not applicable.
